# Assessing upper limb function: transcultural adaptation and validation of the Portuguese version of the Stroke Upper Limb Capacity Scale

**DOI:** 10.1186/s13102-017-0078-9

**Published:** 2017-08-03

**Authors:** João Paulo Branco, Sandra Oliveira, João Páscoa Pinheiro, Pedro L. Ferreira

**Affiliations:** 1 Faculty of Medicine of the University of Coimbra, Coimbra, Portugal; 2 Physical and Rehabilitation Medicine Department, Centro Hospitalar Universitário de Coimbra, Coimbra, Portugal; 3 Physical and Rehabilitation Medicine Department, Centro de Medicina de Reabilitação da Região Centro – Rovisco Pais, Tocha, Portugal; 4 Faculty of Economics, University of Coimbra, Coimbra, Portugal; 5 Centre for Health Studies and Research of the University of Coimbra, Coimbra, Portugal

**Keywords:** Stroke, Stroke Upper Limb Capacity Scale, Upper limb, Disability, Functionality, Hemiparesis

## Abstract

**Background:**

Brachial hemiparesis is one of the most frequent sequelae of stroke, leading to important functional disability given the role of the upper limb in executing activities of daily living (ADL). The Stroke Upper Limb Capacity Scale (SULCS) is a stroke-specific assessment instrument that evaluates functional capacity of the upper limb based on the execution of 10 tasks. The objective of this study is the transcultural adaptation and psychometric validation of the Portuguese version of the SULCS.

**Methods:**

A Portuguese version of the SULCS was developed, using the process of forward-backward translation, after authorisation from the author of the original scale. Then, a multicentre study was conducted in Portuguese stroke patients (*n* = 122) to validate the psychometric properties of the instrument. The relationship between sociodemographic and clinical characteristics was used to test construct validity. The relationship between SULCS scores and other instruments was used to test criterion validity.

**Results:**

Semantic and linguistic adaptation of the SULCS was executed without substantial issues and allowed the development of a Portuguese version. The application of this instrument suggested the existence of celling effect (19.7% of participants with maximum score). Reliability was demonstrated through the intraclass correlation coefficient of 0.98. As for construct validity, SULCS was sensible to muscle tonus and aphasia. SULCS classification impacted the scores of the Motor Evaluation Scale for Upper Extremity in Stroke (MESUPES) and the Stroke Impact Scale (SIS).

**Conclusions:**

The present version of SULCS shows valid and reliable cultural adaptation, with good reliability and stability.

## Background

Stroke continues to be associated with high morbidity and mortality worldwide. In Portugal, the mortality rate from cerebrovascular diseases decreased from 71.9 deaths per 100,000 inhabitants in 2009 to 54.6 deaths per 100,000 inhabitants in 2013 [1]. The majority of strokes were of ischaemic aetiology, occurring mostly in individuals >65 years-old, with atherosclerosis as the main cause [2].

Brain lesions due to cerebrovascular diseases are, currently, one of the leading causes of disability [3]. Approximately 70% of patients that survive stroke present hemiparesis with brachial predominance in the acute phase [4], 30% of those require inpatient care during the first 3 months and, ultimately, 15–30% of patients show permanent disability [5]. Approximately 70% of patients with mild to moderate paresis recover some degree of manual dexterity within the first 6 months, while patients with paresis of the limb with reduced muscle strength do not recover [6].

The upper limb is crucial in executing activities of daily living (ADL) [6] and, therefore, there is a need for an assessment instrument capable of evaluating and quantifying upper limb functionality after stroke. Several instruments have been developed to assess motor, sensitive, and functional ability after stroke [7, 8], including the Jebsen-Taylor Hand Function Test [9], the Box and Block Test [10], and the Frenchay Arm Test [11]. However, all these instruments require some degree of hand functioning and, as such, are not adequate for patients with severe hand disability. Other instruments such as the Research Arm Test [12] and the Upper Limb Motor Assessment Scale [13] evaluate other parameters beyond functional capacity, which hinders the interpretation of functional capacity results [7].

The Stroke Upper Limb Capacity Scale (SULCS) was developed in this context as an easy-to-use, unidimensional, hierarchical, and internally consistent scale that assesses upper limb capacity after stroke. It was the first assessment instrument that included items evaluating both basic upper limb functioning (activities that require reduced or no hand functioning) and more demanding upper limb functioning (activities that require intensive distal functioning) [7, 14].

The objective of this study is the transcultural adaptation and psychometric validation of the Portuguese version of the SULCS in a sample of Portuguese stroke patients.

## Methods

### Study design and sample

This was a multicentre study conducted in the Centre region of Portugal, in patients with history of stroke (both of ischaemic and/or haemorrhagic nature). A total of 122 patients agreed to participate in the study, most of whom had permanent functional disability. The clinical procedures of the study were conducted between May 2014 and April 2015.

All participants were evaluated by healthcare professionals specialised in rehabilitation in the context of stroke or other neurological conditions. Beyond the Portuguese version of SULCS and the sociodemographic questionnaire, the following assessment instruments were used: generic quality of life scale (EuroQol five dimensions questionnaire [EQ-5D]), specific stroke scale (Stroke Impact Scale [SIS]), and specific upper limb functionality scale (Motor Evaluation Scale for Upper Extremity in Stroke [MESUPES]). All these instruments were previously validated for use in the Portuguese population.

The study was conducted according to the precepts of the Declaration of Helsinki and the Oviedo Convention. The study protocol and data collection instruments were approved by the Ethics Committee of the Faculty of Medicine of the University of Coimbra (reference letter 104-CE-2014). This assessment constituted the basis for approval of the study in all participating centres. All participants provided their written informed consent prior to enrolment in the study.

### Measures

#### Stroke upper limb capacity scale

SULCS is the first assessment instrument that includes items evaluating both basic upper limb functioning (activities that require reduced or no hand functioning) and more demanding upper limb functioning (activities that require intensive distal functioning) [7, 14]. This instrument can be used in men and women with both left and right hemiplegia, and in both ischaemic or haemorrhagic stroke [14]. SULCS assessments are short (approximately six minutes) and are based on 10 specific activities that are related to the patients’ ADLs. Three items evaluate proximal functioning (in which there is no active function from the hand and fist), four items evaluate functioning that requires basic control of the fist and fingers, and three items evaluate advanced distal functioning [6, 7, 14].

SULCS assessments can use “start and stop” rules with which it is possible to forgo the assessment of tasks that were previously successfully executed or to interrupt the evaluation when the patient is incapable of executing certain tasks. In this instrument, tasks are unidimensional and hierarchically ordered by increasing difficulty of execution. Scoring is dichotomous: 0 points if it is not possible to execute the task or 1 point if the task is properly executed. The sum of the scores from each task leads to a total score that varies from 0 to 10 points, with higher scores indicating better functioning. Results from SULCS assessments can be summarised in the following categories: “no hand functioning” (0–3 points), “basic hand functioning” (4–7 points), “good hand functioning” (8–10 points) [6]. Contrary to other instruments previously used in this context, SULCS can categorise patients according to their proximal and distal functioning, which provides valuable insight for patient prognosis in terms of functional recovery.

#### EuroQol five dimensions questionnaire

EQ-5D allows the general assessment of health-related quality of life [15]. This instrument evaluates several dimensions: mobility, self-care, usual activities, pain/discomfort, and anxiety/depression. It also includes a self-evaluation component, in which the respondents evaluate their overall health status through a visual analogue scale (EQ-VAS), ranging from 0 (worse health status imaginable) to 100 (best health status imaginable). The Portuguese validated version of EQ-5D and the corresponding valuing system were used in this study [16, 17].

#### Stroke impact scale

SIS is a stroke-specific instrument designed to assess multidimensional stroke outcomes; it consists of 65-items grouped in the following categories: strength (four items), memory and thinking (eight items), emotion (nine items), communication (seven items), participation (12 items), mobility (10 items), hand function (five items), ADLs/instrumental ADLs (9 items), and recovery (1 item) [18, 19]. The Portuguese validated version of SIS was used in this study [19].

#### Motor evaluation scale for upper extremity in stroke (MESUPES)

MESUPES was designed to evaluate the quality of upper limb movements after stroke. It consists of 17 items, 8 of which refer to proximal upper limb functioning and 9 to distal upper limb functioning [20, 21].

### Translation and transcultural adaptation

SULCS was developed in 2001 at the Sint Maartenskliniek Rehabilitation Centre, in Nijmegen, The Netherlands. Between 2001 and 2008, SULCS was applied to 546 patients hospitalised in this centre and in 2010 the data from these assessments were used to evaluate the reliability, sensitivity, and validity of the scale. The results of this analysis revealed that SULCS has outstanding psychometric properties [7]. The scale has since been translated and validated for the English language and a French validation is in progress.

After authorisation from the author of the original SULCS, a Portuguese version of the scale was developed, using the process of forward-backward translation [22]. The process of forward translation consisted of an initial preparation of two translations, completed by two independent Portuguese bilingual translators, one translator was a professional translator and the other was a PhD and teacher of English. Then, a consensus version was prepared by two of the authors (PLF and JPB). This consensus version was then back-translated by a native English translator. The back-translated version was evaluated by the authors PLF, JPB, and JPP to verify agreement with the original instrument and, then, a final version of the Portuguese SULCS translation was prepared. Subsequently, a Portuguese expert in the field of rehabilitation conducted a clinical review to obtain a more specialised translation, which would be easier to understand by employing language with which the patients were more familiar.

### Statistical analyses

The acceptability of the scale was tested through the quantification of missing data and the distribution of the sample by the various categories of the scale. The following hypothesis was formulated:

- H_1_: The SULCS scale presents no significant missing data and its distribution is spread over the various categories.

Concordance between observers was evaluated in 41 participants. Each of those participants was subject to two evaluations by different healthcare professionals. Cohen’s kappa coefficient and the criteria defined by Landis and Koch were used for this analysis [15].

To test the construct validity of SULCS, criteria derived from sociodemographic and clinical variables in known samples were used. The following hypotheses were tested:

- H_2_: Patient gender impacts SULCS score.
- H_3_: Patient age impacts SULCS score.
- H_4_: Stroke aetiology (haemorrhagic/ischaemic) impacts SULCS score.
- H_5_: Left/right lateralisation of the affected limb impacts SULCS score.
- H_6_: Tonus changes impact SULCS score.
- H_7_: Presence of communication impairment (aphasia) impacts SULCS score.

These hypotheses were tested using Chi-square tests, as well as tests for the comparison of means (*t*-Student for two independent samples or ANOVA for more than two samples). *Post-hoc* multiple comparison analyses were used when applicable.

To test criterion validity, SULCS was categorised into “no hand functioning” (0–3 points), “basic hand functioning” (4–7 points), and “good hand functioning” (8–10 points), and then compared with EQ-5D, SIS, and MESUPES, with the objective of testing the following hypotheses:

- H_8_: Patients with good hand functioning according to SULCS show higher EQ-5D scores.
- H_9_: Good hand functioning according to SULCS is highly associated with SIS.
- H_10_: SULCS scores are highly associated with MESUPES total scores.

Statistical analyses were performed with IBM SPSS Statistics for Windows, Version 23.0. (IBM Corp, Armonk, NY, USA). A 5% level of significance was adopted.

## Results

### Semantic and linguistic adaptation

The changes suggested after the expert review were scarce and based on clinical and linguistic criteria. In the original SULCS instrument there was a reference to the term “affected forearm”, which had been equivalently translated and was changed during the expert review to the Portuguese equivalent of “affected limb”. The remaining changes consisted of verb conjugation corrections. Therefore, content validity was obtained.

### Study sample

Data obtained from the sociodemographic questionnaire of the 122 participants are presented in Table 1. Mean age in the study sample was 67.8 ± 12.1 years and mean time after stroke was 14.0 months. There was no significant predominance in terms of gender or laterality. Most participants had had stroke of ischaemic origin, and most showed no discernible spasticity or aphasia.

**Table 1:** Demographic and clinical characteristics of the study population

Variable	Study population (*n* = 122)
Gender, n (%)	
Male	65 (53.3)
Female	57 (46.7)
Age (years)	
Mean ± standard deviation	67.8 ± 12.1
Min	32
Max	94
Time after stroke (months)	
Mean ± standard deviation	14.0 ± 16.5
Median (min, max)	10.3 (1.6, 131.0)
Trimmed Mean 5%	11.4
Type of stroke, n (%)	
Ischaemic	81 (66.4)
Haemorrhagic	25 (20.5)
Undetermined	16 (13.1)
Laterality, n (%)	
Right	56 (45.9)
Left	65 (53.7)
Tonus, n (%)	
Spastic	28 (23.0)
Aphasia, n (%)	
Yes	38 (32.1)

Table 2 presents the descriptive statistics for the various assessment instruments used in the study. Perception of health-related quality of life was fairly low in the study population, as suggested by the mean EQ-5D score of 0.21. This value decreased with advancing age: 0.33 (<50 years), 0.27 (50–64 years), 0.18 (65–74 years), and 0.15 (≥75 years).

**Table 2:** Descriptive statistics for the EQ-5D, SIS, SULCS, and MESUPES scales in the study population (*n* = 122)

Variable	Minimum	Maximum	Mean	Standard deviation
EQ-5D				
Dimensions	−0.49	1.0	0.21	0.3
EQ-VAS	20.0	90.0	51.7	16.5
SIS				
Recovery	10.0	95.0	53.0	20.4
Strength	12.5	100.0	41.2	19.1
Memory	0.00	100.0	73.4	25.1
Emotion	33.3	80.6	57.7	10.5
Communication	0.00	100.0	80.2	24.6
Activities of daily living	4.2	100.0	45.8	24.7
Mobility	2.5	100.0	49.8	27.5
Hand function	0.0	100.0	32.4	33.0
Participation	11.1	100.0	53.9	20.4
SULCS	0.0	10.0	6.0	3.3
MESUPES				
Hand	0.0	2.0	47.9	36.9
Shoulder	0.0	5.0	73.9	33.0
Total	0.0	3.4	65.6	32.7

*EQ-5D* EuroQol five dimensions questionnaire, *MESUPES* Motor Evaluation Scale for Upper Extremity in Stroke, *SIS* Stroke Impact Scale, *SULCS* Stroke Upper Limb Capacity Scale

According to SIS scores, the dimensions that showed higher impairment after stroke in this population were hand function (32.4%), strength (41.2%), and ADLs (45.8%). Communication was the dimension evaluated by SIS in which participants showed better results.

Mean SULCS score was 6.0. Most participants (43.3%) showed good hand functioning, 24.6% showed basic hand functioning, and 32.0% showed no hand functioning.

In the affected limb, according to MESUPES scores, there was greater functional impairment at the hand rather than the shoulder (47.9% and 73.9%, respectively).

### Acceptability and distribution

Since SULCS is applied by healthcare professionals, there were no comprehension issues or any other difficulty in conducting the assessments; this is reflected by the absence of missing data (Hypothesis H_1_). The distribution of the sample included all SULCS categories: no hand function (32.0%), basic hand functioning (24.6%), and good hand functioning (43.3%). However, the highest SULCS score (10) was registered in 24 patients (19.7%), thus showing evidence of a ceiling effect (according to a 15% criterion).

### Reliability

SULCS showed a 0.984 intraclass correlation coefficient. Since SULCS allows categorisation (no hand functioning, basic hand functioning, and good hand functioning), a 0.886 Cohen’s kappa coefficient was also calculated.

### Construct validity

Construct validity was tested by assessing how different values of sociodemographic and clinical characteristics impacted SULCS scores, as shown in Table 3. When considering hypotheses H_2_ to H_7_, only the variables change in muscle tonus and the presence of aphasia were determinants for hand functioning as measured by SULCS (Hypothesis H_6_).

**Table 3:** Mean SULCS scores according to sociodemographic and clinical characteristics of the study population

Variable	Value	N	Mean ± SD	| *t* |	*p*-value
Gender	Male	65	6.15 ± 3.22	0.698	0.486
	Female	57	5.74 ± 3.37		
Age	<65 years old	50	5.90 ± 3.30	0.165	0.869
	≥65 years old	72	6.00 ± 3.29		
Type of stroke	Ischaemic	81	5.93 ± 3.37	0.061	0.952
	Haemorrhagic	25	5.88 ± 3.14		
Laterality	Right	56	6.01 ± 3.51	0.081	0.936
	Left	65	5.97 ± 3.09		
Tonus	Spastic	28	3.32 ± 2.68	5.265	<0.001
	Non-spastic	79	6.79 ± 3.09		
Aphasia	Yes	38	4.75 ± 3.44	2.533	0.013
	No	84	6.44 ± 3.13		

*SD* Standard deviation, *SULCS* Stroke Upper Limb Capacity Scale

### Criterion validity

To test criterion validity, SULCS scores (in categorised from) were compared with scores from EQ-5D, SIS, and MESUPES, as shown in Table 4. That is, we tested whether different functional categories given by SULCS correspond to different average scores given by EQ-5D, SIS, and MESUPES.

**Table 4:** Mean EQ-5D, SIS, and MESUPES scores according to SULCS scores (in categorised form)

	SULCS category					
Variable	No hand functioning [1]	Basic hand functioning [2]	Good hand functioning [3]	*F*	*p*-value	Post-hoc multiple comparison
EQ-5D						
Dimensions	0.01 ± 0.29	0.20 ± 0.25	0.36 ± 0.24	20.75	<0.001	1 < 2 < 3
EQ-VAS	48.2 ± 14.6	47.7 ± 17.4	56.6 ± 16.2	4.32	0.015	1 = 2 < 3
SIS						
Recovery	46.1 ± 17.9	48.5 ± 20.2	60.6 ± 20.1	7.29	0.001	1 = 2 < 3
Strength	27.4 ± 12.9	39.6 ± 15.9	52.4 ± 17.7	27.95	<0.001	1 < 2 < 3
Memory	74.0 ± 24.8	69.0 ± 30.5	75.5 ± 22.2	0.66	0.516	
Emotion	57.6 ± 10.3	55.2 ± 11.1	59.3 ± 10.3	1.46	0.236	
Communication	77.4 ± 27.8	79.4 ± 27.8	82.7 ± 20.0	0.54	0.586	
ADL	29.7 ± 18.9	40.7 ± 22.2	60.5 ± 21.3	26.01	<0.001	1 < 2 < 3
Mobility	32.8 ± 23.4	46.6 ± 25.7	64.2 ± 23.5	19.53	<0.001	1 < 2 < 3
Hand function	2.1 ± 5.1	23.5 ± 22.4	59.8 ± 26.9	87.06	<0.001	1 < 2 < 3
Participation	45. 9 ± 15.8	49.9 ± 21.5	62.1 ± 20.1	8.96	<0.001	1 = 2 < 3
MESUPES						
Hand	0.05 ± 0.14	0.45 ± 0.3	0.79 ± 0.2	180.52	<0.001	1 = 2 < 3
Shoulder	0.33 ± 0.24	0.84 ± 0.1	0.99 ± 0.0	205.52	<0.001	1 = 2 < 3
Total	0.24 ± 0.19	0.72 ± 0.1	0.93 ± 0.1	289.65	<0.001	1 = 2 < 3

*EQ-5D* EuroQol five dimensions questionnaire, *MESUPES* Motor Evaluation Scale for Upper Extremity in Stroke, *SIS* Stroke Impact Scale, *SULCS* Stroke Upper Limb Capacity Scale

Participants with good hand functioning according to SULCS showed better overall health status, as measured through EQ-5D (mean EQ-5D of 0.36), than the remaining groups (Hypothesis H_8_).

All dimensions of SIS were significantly impacted by SULCS scores (Hypothesis H_9_); there were particularly strong effects for the dimensions of recovery, ADLs, mobility, hand function, and participation (all *p* < 0.001). For all these dimensions, participants with good hand functioning showed higher scores in SIS, as compared to the remaining groups. For the dimensions of memory, emotion, and communication, as would be expected, the effects were of lesser magnitude.

The MESUPES scores were also impacted by SULCS scores, including both dimensions and the total score (Hypothesis H_10_).

## Discussion

Stroke has a reported incidence of 1.9% in the general population, but is more frequent in men and individuals with age 65 to 74 years-old [23]. Ischaemic aetiologies represent the majority of strokes (87%, thrombotic or embolic), while haemorrhagic aetiologies represent a small fraction of cases (13%, intracerebral or subarachnoid) [24]. In this study, the mean age was over 65 years and there was a predominance of ischaemic stroke, as expected according to the literature. However, we found no statistically significant differences in terms of gender predominance or laterality. It should be highlighted that there was a mean time from stroke of approximately 14 months and spasticity and aphasia were not particularly clinically relevant in this population.

Stroke continues to be one the main causes of health-related quality of life impairment in the country [25], which is reflected in the EQ-5D scores from this sample (EQ-5D of 0.21). These findings are consistent with previous studies, and further highlight the medical and social impact of stroke in the Portuguese population. However, despite the impact of stroke in the Portuguese population there is not yet a validated instrument to specifically assess upper limb functioning in these patients. The development of such functional evaluation tools is crucial to improve the clinical management of stroke patients and to allow the development of clinical research in rehabilitation medicine. SULCS is an easy-to-use, unidimensional, hierarchical, and internally consistent scale that evaluates upper limb functioning after stroke and can provide valuable insight in both clinical practice and clinical research contexts.

SIS scores were significantly impacted by SULCS classification for all dimensions, with particularly stronger impact for the dimensions of strength, ADLs, mobility, hand function, and participation. These findings can be explained by the fact that most of the tasks alluded by SIS need proper upper limb function to be adequately executed. The recovery dimension was also impacted by the SULCS classification of participants, which highlights the importance of upper limb functional recovery for general recovery after stroke. SULCS classification did not, however, impacted the dimensions of memory, emotion, and communication, as would be expected since these aspects are not particularly dependent on upper limb functioning.

MESUPES scores were also strongly impacted by SULCS classification for both dimensions as well as the total score. Such findings were expected, since SULCS and MESUPES evaluate highly-related types of functioning and, therefore, these results confirm the capacity of this version of SULCS in establishing upper limb functionality.

In terms of EQ-5D results, this population showed substantially impaired quality of life, but, interestingly, average SULCS scores were only indicative of moderate upper limb functional impairment (with mean SULCS score within the basic hand functioning category) [6]. These findings highlight the importance of using specific instruments to assess the several domains that can impact a patient’s quality of life and health status, since general quality of life assessments do not always accurately represent aspects that play important roles in performing ADL’s, such as upper limb functioning.

Several factors associated with worse prognosis after stroke have been described in the literature, including advanced age, right hemisphere involvement, lesions of haemorrhagic nature, and cognitive impairment [26–28]. In this study, however, there were no statically significant associations between age, gender, aetiology, and laterality and upper limb functional capacity as measured by SULCS. These results can be explained by the fact that SULCS is a highly-specific instrument aimed at evaluating upper limb functioning and not overall patient functionality or health status (as assessed in previous studies). As for the relationship between gender and functional recovery after stroke, this study is in agreement with previous evidence, indicating that gender is not significantly associated with functional prognosis [26, 28].

The presence of spasticity in the upper limbs after stroke has been associated with impaired functionality and high levels of dependence [29]. In this study, patients with higher degree of spasticity showed lower upper limb functional capacity (with significantly lower SULCS scores), as expected. The difference in SULCS scores for patients showing spasticity vs. non-spastic patients was highly significant (*P* < 0.001), despite the fact that only 23% of the study population showed spasticity in the upper limbs, thus, further emphasising the relevance of this difference. Further studies should explore the effect of spasticity on long-term upper limb functioning as measured by SULCS.

Patients with aphasia showed significantly lower upper limb functional capacity according to SULCS scores. In this case, however, the findings should be interpreted with caution, since there is a potential for bias due to the communication deficits of patients with aphasia and the inherent difficulties in applying the assessment instrument.

This study has limitations that should be considered. The study population was relatively heterogeneous (particularly in terms of age distribution and time since acute stroke). The multicentre design has some disadvantages to consider, particularly the larger number of healthcare professionals involved in the study procedures, which may increase the potential for subjectivity in the assessments. Patients with aphasia were enrolled, including those with expression aphasia, which might pose a bias in interpreting the results from assessment scales. The population also had substantially impaired overall health status, which can bias the results towards lower quality of life and impaired functioning.

## Conclusions

The process of translation and transcultural adaptation of SULCS to the Portuguese population was performed without substantial issues, with high concordance between translation and expert review.

In terms of sociodemographic and clinical factors, the instrument was sensible to muscle tonus and aphasia. There was high concordance between observers, which demonstrates reliability.

Health-related quality of life decreased with advancing age in this patient population, but this factor was not reflected in hand functioning.

SULCS classification impacted MESUPES and SIS dimensions, with stronger impacts in the dimensions of strength, ADLs, mobility, hand function, and participation. These data provided the basis to confirm both construct and criterion validity.

Therefore, the present version of SULCS is validated for use in the Portuguese population.

## Abbreviations

ADL: Activities of daily living
EQ-5D: EuroQol five dimensions questionnaire
MESUPES: Motor evaluation scale for upper extremity in stroke
SIS: Stroke impact scale
SULCS: Stroke upper limb capacity scale
VAS: Visual analogue scale
